# Blindness in New-Born Infants

**Published:** 1891-06

**Authors:** 


### BLINDNESS IN NEW-BORN INFANTS.

There are, says Mr. S. Snell, Ophthalmic Surgeon to the Sheffield General Infirmary and to the Institution for the Blind, few things more sad in my experience than to witness infants of only a week or two old, brought for treatment, with one or both eyes either already lost or irreparably damaged. Nor is this feeling rendered less by the reflection that the disease which has led to such dire results could have been in the first instance prevented, or, if that opportunity had passed, timely treatment would have brought about recovery of sight. A card bearing the following caution has been distributed among parents bringing children to the general infirmary:—"Important. If a baby's eyes run with matter and look red a few days after birth, take it at once to a doctor. Delay is dangerous, and one or both eyes may be desrtoyed if not treated immediately."

